# Quiste estromal primario de iris: a propósito de un caso

**DOI:** 10.23938/ASSN.1072

**Published:** 2024-05-17

**Authors:** Inés Munuera, Jorge Sánchez-Monroy, Martín Puzo, Antonio Mateo, Silvia Méndez-Martínez

**Affiliations:** 1 Servicio Aragonés de Salud Hospital Universitario Miguel Servet Departamento de Oftalmología Zaragoza España; 2 Servicio Aragonés de Salud Hospital San Jorge Departamento de Oftalmología Huesca España

**Keywords:** Enfermedades del Iris, Quiste, Segmento Anterior del Ojo, Tomografía de Coherencia Óptica, Tratamiento, Iris Diseases, Cysts, Diagnostic imaging, Anterior eye segment, Tomography, Optical Coherence, Management

## Abstract

Los quistes estromales primarios de iris son poco comunes, a menudo asintomáticos y de detección incidental; su tratamiento está indicado en casos que presentan crecimiento o complicaciones. Sin embargo, requieren pruebas de imagen para determinar su naturaleza quística y hacer un diagnóstico diferencial preciso con tumores malignos, así como para realizar el seguimiento a lo largo del tiempo. La biomicroscopía ultrasónica es la técnica de elección, pero la tomografía de coherencia óptica de segmento anterior (OCT-SA) es una prueba más accesible y disponible en la mayoría de los centros.

Se presenta un caso de quiste estromal primario de iris de presentación atípica para ilustrar el diagnóstico y seguimiento inicial mediante OCT-SA y fotografías, así como el manejo de las complicaciones. La OCT-SA podría tener su utilidad en el estudio inicial y seguimiento de lesiones anteriores, no pigmentadas y en las que se visualice el quiste en su totalidad.

## Abstract (en)

Primary stromal cysts of the iris are rare, often asymptomatic, and incidentally found entities. Treatment is usually indicated in cases of enlargement or complications. However, imaging tests are required to determine their cystic nature and make an accurate differential diagnosis with malignant tumors, as well as for long-term follow-up. Ultrasound biomicroscopy is the technique of choice, although in most centers anterior segment optical coherence tomography is a more accessible and available imaging modality.

We present a case of primary stromal cyst of the iris with an atypical presentation to illustrate the diagnosis and initial follow-up using anterior segment optical coherence tomography and photographs, and the management of complications. Anterior segment optical coherence tomography may be useful in the initial study and follow-up of anterior non-pigmented lesions where the cyst can be fully seen.

## INTRODUCCIÓN

Pese a ser uno de los tumores más frecuentes de segmento anterior, los quistes de iris son una patología poco común^1^.

Los quistes primarios no tienen una etiología definida, aunque se postula que pueden deberse a un defecto del desarrollo neuroectodérmico^2^. Se clasifican en quistes del epitelio pigmentario del iris (producidos por desdoblamiento de la bicapa de epitelio, con contenido seroso claro) o estromales (por crecimiento epitelial en el propio estroma, menos frecuentes)^3^. Los quistes secundarios se originan por la entrada de células epiteliales de la superficie, y entre sus causas desatacan los traumatismos, penetrantes o quirúrgicos, y el uso prolongado de agentes mióticos o prostaglandinas^1^.

A menudo son asintomáticos y pueden detectarse de manera casual en el estudio biomicroscópico. Sin embargo, el diagnóstico diferencial con tumores malignos de segmento anterior debe ser preciso, así como el manejo adecuado de las posibles complicaciones a largo plazo, por lo que se requieren pruebas de imagen para caracterizar de forma detallada la lesión^1^. Se han publicado múltiples estudios comparando la tomografía de coherencia óptica de segmento anterior (OCT-SA) y la biomicroscopía ultrasónica (BMU)^4^^-^^7^. La BMU es la técnica de elección^5^ porque la OCT-SA tiene limitada penetrancia a través del tejido pigmentario y la visualización del tumor completo y la definición del margen posterior son inferiores. Sin embargo, la OCT-SA es una prueba más accesible, presente en la mayoría de los centros.

Se presenta un caso de un quiste primario estromal de iris de presentación atípica para ilustrar el diagnóstico y seguimiento inicial mediante OCT-SA (como alternativa a la BMU) y fotografías, así como el manejo de las complicaciones.

## CASO CLÍNICO

Se presenta el caso de un varón de 60 años, con antecedentes de paraplejia postraumática y tricoleucemia tratada con quimioterapia, que acudió a urgencias por sensación de cuerpo extraño y lagrimeo en ojo derecho de dos semanas de evolución. Negaba traumatismo previo o antecedentes de cirugía ocular.

**Figura 1:**
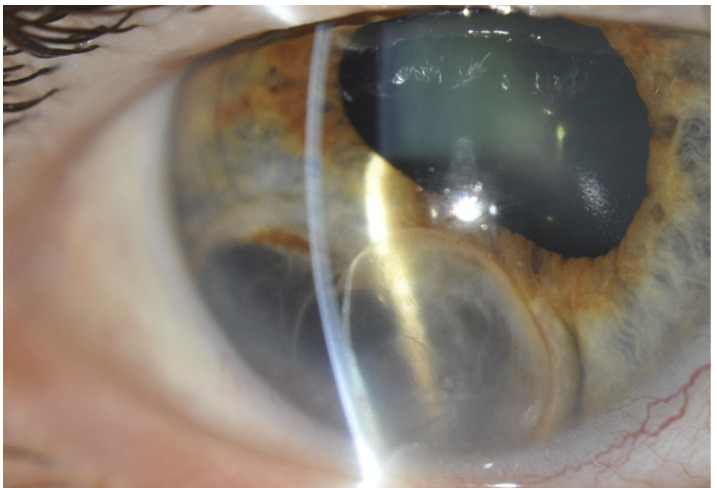
Fotografía de segmento anterior del ojo derecho. Se observa en el sector inferotemporal del iris una formación quística bilobulada de contenido transparente que protruye anteriormente y transilumina por atrofia del epitelio pigmentario del iris subyacente.

A la exploración se observó una agudeza visual de 0,7 que mejoraba a 1 con estenopeico en ojo derecho y unidad en ojo izquierdo. Se detectó anisocoria con ambas pupilas reactivas, por corectopia del ojo derecho. Con lámpara de hendidura se observó en el sector inferotemporal del iris derecho una formación quística bilobulada, de contenido transparente, que protruía anteriormente y transiluminaba por atrofia del epitelio pigmentario del iris subyacente (Figura 1). No se asociaba a catarata sectorial. La presión intraocular era de 16 mm Hg en ambos ojos y mediante gonioscopía no se observó cierre angular. El polo posterior resultó normal.

**Figura 2:**
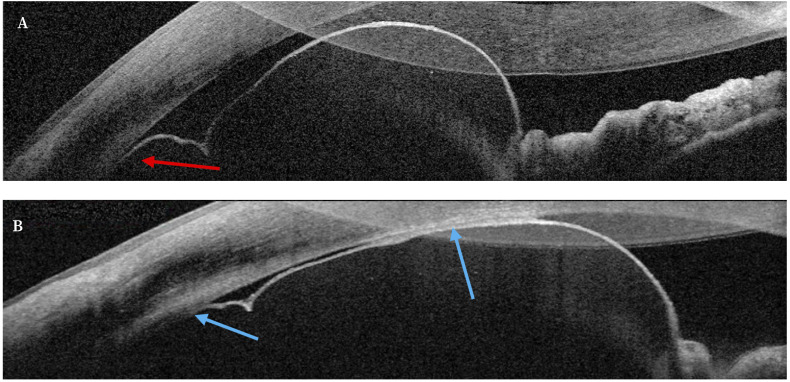
Tomografía de coherencia óptica de segmento anterior. Masa bilobulada de pared anterior fina e integridad del epitelio pigmentario del iris, con contenido líquido, compatible con quiste estromal de iris. A. Al diagnóstico, mínimo contacto con el endotelio corneal en periferia inferior (flecha roja). B. Contacto endotelial aumentado (fechas azules) tras un mes de seguimiento.

Ante la falta de BMU, se realizó una OCT-SA donde se apreció una masa bilobulada de pared anterior fina e integridad del EPI con contenido líquido, compatible con un quiste estromal de iris, y con contacto mínimo con el endotelio corneal en periferia inferior (Figura 2A). Se realizó una radiografía simple craneal para descartar la presencia de cuerpos extraños.

Ante la ausencia de signos de malignidad, y dado que la presión intraocular era normal, el contacto endotelial mínimo y no había signos de inflamación, se decidió observación periódica para vigilar la aparición de estas posibles complicaciones. Al mes de seguimiento se observó en la OCT-SA un crecimiento de la lesión con aumento de contacto endotelial (Figura 2B).

Debido al riesgo de sufrir una descompensación corneal, se decidió realizar tratamiento con láser argón para liberar tensión de las paredes y minimizar así la superficie de contacto.

**Figura 3:**
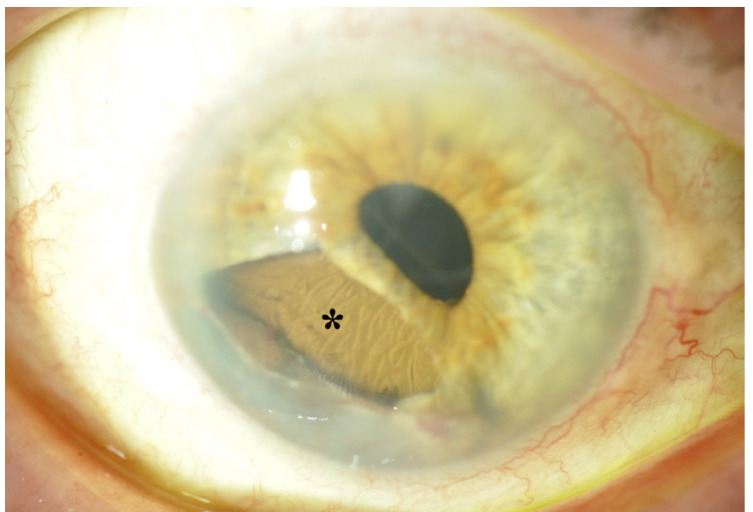
Fotografía de polo anterior tras la cirugía e implantación del fragmento de iris artificial (asterisco).

A los seis meses de seguimiento, los impactos de láser en iris presentaban una cicatrización correcta y el contacto endotelial, periférico, era mínimo. Sin embargo, y junto a la recidiva del quiste, se constató la aparición de una facoesclerosis nuclear grado 3 subluxada que generaba un astigmatismo lenticular, por lo que se indicó tratamiento quirúrgico. Se realizó cirugía de facoemulsificación con implante de lente intraocular en saco, iridectomía sectorial de la zona afectada por el quiste con preservación del esfínter pupilar del iris, e implante de una porción de iris artificial de 9 mm sobre sulcus. (Figura 3).

El resultado final fue estética y funcionalmente satisfactorio. Durante el seguimiento del paciente no se ha detectado crecimiento de la lesión ni aparición de nuevas complicaciones.

## DISCUSIÓN

Los quistes de iris a menudo presentan dificultades diagnósticas y terapéuticas. Los quistes secundarios requieren una historia detallada sobre traumatismos, cirugía ocular, uso de fármacos o tumores malignos sistémicos para determinar si existe una etiología asociada^1^^,^^9^. Este caso es de presentación atípica por la ausencia de antecedentes de interés y por la edad del paciente, puesto que los quistes primarios suelen ser congénitos, aunque lo más común es que se manifiesten en la adolescencia^1^.

La exploración ha de ir dirigida a identificar y caracterizar la lesión y descartar complicaciones asociadas. Debe incluir examen de agudeza visual, biomicroscopía, presión intraocular y gonioscopía, si bien el diagnóstico de certeza para determinar su naturaleza quística y realizar un diagnóstico diferencial con tumores de segmento anterior requiere técnicas de imagen, siendo la BMU la técnica de elección^5^. Ante la falta de disponibilidad de BMU en nuestro servicio, se realizó una OCT-SA. Teniendo en cuenta sus limitaciones y desventajas, las características de la lesión permitieron dar por válida la prueba.

Los quistes de iris a menudo son asintomáticos y permanecen estables, lo que generalmente permite su observación^3^^,^^8^^,^^9^. El tratamiento se reserva para casos que presentan crecimiento o complicaciones, tales como descompensación corneal, obstrucción del ángulo iridocorneal con el consecuente aumento de presión intraocular y glaucoma secundario, inflamación en forma de uveítis anterior u obstrucción del eje visual^8^^,^^9^. El riesgo de complicaciones es mayor en los quistes primarios estromales y en los secundarios traumáticos^3^^,^^8^. En el momento del diagnóstico inicial, el paciente del presente caso no presentaba signos de malignidad ni de complicaciones (mínimo contacto endotelial), por lo que se adoptó una estrategia de observación. No obstante, al mes de seguimiento se observó crecimiento de la lesión acompañado de un aumento de contacto endotelial. Ante el riesgo de descompensación corneal, se consideró que el paciente era candidato a recibir tratamiento.

La elección de la técnica entre la amplia variedad de posibilidades terapéuticas para el manejo de los quistes de iris dependerá de las características del escenario clínico.

Para disminuir el riesgo de complicaciones iatrogénicas, la tendencia actual es elegir opciones mínimamente invasivas^1^^,^^8^. La aspiración con aguja fina, aunque ha logrado la regresión permanente de algunos quistes de iris, generalmente requiere la combinación con otras modalidades terapéuticas^10^^,^^11^, por lo que su utilidad reside en el diagnóstico de tumores potencialmente malignos no metastáticos cuando otros métodos no son efectivos^1^. La esclerosis con etanol puede reducir o estabilizar el tamaño de un quiste de iris, aunque en algunos casos se requiere repetir el procedimiento^12^^,^^13^. La inyección de agentes antimitóticos, como la mitomicina-C o el 5-fluorouracilo, ha logrado respuesta favorable en algunos casos^14^^,^^15^, lo que podría tener su utilidad en el tratamiento de quistes recurrentes o resistentes a otras formas de terapia. La fotocoagulación con láser Argón o la fotodisrupción con láser Neodinio:YAG (Nd:YAG) del epitelio permiten detener la producción de líquido intraquístico o destruir gradualmente el quiste mediante aplicaciones repetidas, disminuyendo el riesgo de lesionar estructuras circundantes. Constituye un enfoque mínimamente invasivo que resulta especialmente útil en pacientes con riesgo quirúrgico, niños o casos con riesgo aumentado de complicaciones, y que cuenta con resultados favorables^16^^,^^17^.

La cirugía es efectiva pero tiene sus complicaciones, por lo que debe ser considerada como último recurso, generalmente tras el fracaso de otras opciones menos invasivas, o como primera indicación si las características de la lesión anticipan su necesidad o ante la sospecha de malignidad, ya que permite obtener muestras de tejido. Existen gran variedad de abordajes quirúrgicos, desde legrado corneal hasta escisión simple o asociada a otras estructuras, según la extensión y naturaleza del quiste^8^.

En nuestro caso, tras iniciar el tratamiento mediante fotocoagulación con láser Argón, ante la recidiva del quiste y la aparición de complicaciones, se indicó tratamiento quirúrgico. Se realizó escisión del quiste con iridectomía sectorial, preservando el esfínter del iris para evitar sintomatología de deslumbramientos, asociado a facoemulsificación e implantación de lente en saco y colocación de una porción de iris artificial sobre *sulcus*, posterior a iris, para evitar también deslumbramientos en la zona descubierta por la iridectomía sectorial.

En conclusión, los quistes estromales primarios de iris representan una patología ocular poco común que, aunque frecuentemente asintomática y detectada de forma incidental, requiere de un diagnóstico diferencial meticuloso para excluir tumores malignos y de un seguimiento cuidadoso para identificar potenciales complicaciones. Este caso destaca la utilidad de la OCT-SA como una herramienta diagnóstica alternativa accesible, especialmente en contextos donde la BMU no esté disponible, para el estudio inicial y el seguimiento longitudinal de lesiones anteriores, no pigmentadas, en las que el quiste se visualiza en su totalidad.

A través del caso clínico presentado se ilustra no solo la aplicación de esta tecnología en la práctica clínica, sino también el manejo de las complicaciones surgidas, resaltando la importancia de una estrategia terapéutica adaptativa que priorice intervenciones mínimamente invasivas y mantenga el tratamiento quirúrgico como una opción ante casos de crecimiento del quiste o complicaciones.
